# Association between changes in blood eosinophil levels and alterations in olfactory function in asthma

**DOI:** 10.1186/s13223-026-01025-1

**Published:** 2026-03-28

**Authors:** Hyo-In Rhyou, Woo Yong Bae, Young-Hee Nam

**Affiliations:** 1 Department of Internal Medicine, Inje University Haeundae Paik Hospital, Inje University College of Medicine, Busan, Republic of Korea; 2 Department of Otorhinolaryngology-Head and Neck Surgery, Dong-A University, Busan, Republic of Korea; 3 Department of Internal Medicine, College of Medicine, Donga-A University, 26-Daesingongwon-ro, Seo-Gu, Busan, Republic of Korea

**Keywords:** Asthma, Olfaction, Eosinophils, Blood

## Abstract

Olfactory dysfunction has been observed in asthma, but its underlying mechanisms are poorly understood. This prospective study assessed olfactory function using the YSK Olfactory Function Test in 30 patients with moderate to severe asthma, and compared clinical parameters according to changes in olfactory function over 6 months. Nine patients were classified into the olfactory-improvement group, 15 into the olfactory-stable group, and six into the olfactory-decline group. The change in blood eosinophil levels showed a progressive increase across the olfactory-improvement, olfactory-stable, and olfactory decline groups, while there were no significant differences in terms of the presence of upper airway diseases, use of intranasal steroids, asthma control status, and asthma treatment medications among the three groups. Eosinophilic inflammation in blood may be associated with olfactory function in asthma, and further study will be needed in the future.

Olfaction significantly impacts quality of life and can be impaired by various factors, [1] including neurodegenerative disease, [2] upper airway diseases, [2] and chronic conditions such as diabetes mellitus, [3] chronic kidney disease, [4] and chronic obstructive pulmonary disease [5]. Although olfactory dysfunction has been reported in asthma, [6] this association is often overlooked due to the high prevalence of upper airway diseases. This study assessed olfactory function in asthma patients and explored underlying mechanisms.

This prospective observational cohort study enrolled adults (≥ 18 years) with moderate-to-severe asthma who had been treated for at least one year. Moderate-to-severe asthma was defined as requiring medium- or high-dose inhaled corticosteroids plus step 4–5 maintenance therapy according to GINA guidelines. Patients receiving short-term systemic corticosteroids for three or more days within a month for asthma exacerbations or other causes were excluded. All participants continued standard therapy, and biologic therapy was also permitted.

A total of 30 patients were enrolled, and all participants underwent evaluation of clinical asthma parameters and olfactory function at baseline and after six months.

Olfactory function was assessed using the YSK Olfactory Function Test (YOF), which utilizes odors culturally familiar to Koreans and has diagnostic efficacy equivalent to that of the Korean version of the Sniffin’ Stick Test [7]. The YOF total score ranges from 1 to 36, with diagnostic cutoffs of ≤ 14.5 for anosmia and ≤ 21.0 for hyposmia. Participants were categorized based on changes in olfactory status over the six-month period in the following groups:

- *Olfactory-improvement group*: Patients whose olfactory status improved from hyposmia to normosmia or from anosmia to hyposmia or normosmia.
- *Olfactory-stable group*: Patients whose olfactory status (normosmia, hyposmia, or anosmia) remained unchanged over the six-month period.
- *Olfactory-decline group*: Patients whose olfactory status worsened from normosmia to hyposmia or anosmia, or from hyposmia to anosmia.

Statistical analyses were performed using SPSS 22.0 (IBM Corp., Armonk, NY, USA). Continuous variables are presented as mean ± standard deviation and were compared using one-way analysis of variance (ANOVA). Categorical variables are presented as counts and were compared using the chi-square test or Fisher’s exact test. A *P*-value < 0.05 was considered statistically significant.

This study was approved by the institutional review board of Dong-A University Hospital, Busan, Republic of Korea (DAUHIRB-21-155).

In this study, nine patients were classified into the olfactory-improvement group, 15 into the olfactory-stable group, and six into the olfactory-decline group (Fig. 1A). At baseline, 10 patients had normosmia. Among these, two experienced a decline to anosmia after six months, while the remaining eight maintained their olfactory status. Of the 13 patients with hyposmia at baseline, four exhibited no change in olfactory function, five improved to normosmia, and four worsened to anosmia. At baseline, seven patients had anosmia. Among these, three continued to have anosmia, two improved to hyposmia, and two improved to normosmia.

**Fig 1.**
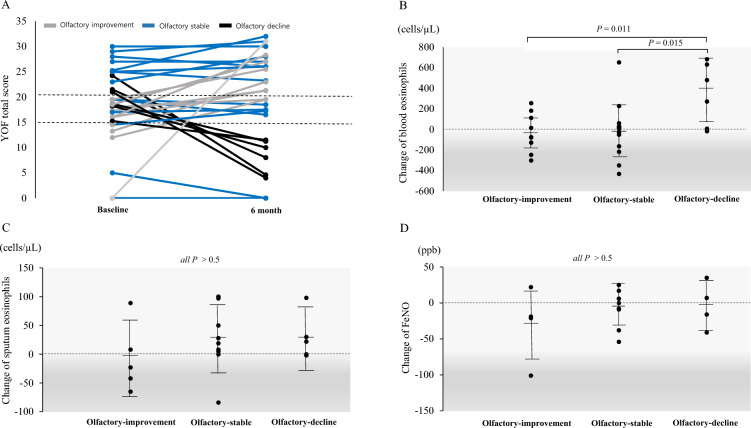
Changes in YOF scores (**A**), blood eosinophil levels (**B**), sputum eosinophil levels (**C**), and FeNO levels (**D**) among the olfactory-improvement, olfactory-stable, olfactory-decline groups. In Fig. 1A, gray line implies the olfactory-improvement group, blue line implies the olfactory-stable group, and black line implies the olfactory-decline group. FeNO, fractional exhaled nitric oxide; YOF, YSK Olfactory Function test

There were no significant differences among the three groups in sex, age, smoking status, presence of severe asthma, or incidence of asthma exacerbations before or during the study period. No significant differences were observed in the presence of allergic rhinitis (AR), chronic rhinosinusitis (CRS) with or without nasal polyp (NP) or a history of sinus surgery, and the use of intranasal steroids during the study period among the three groups (Table 1).

**Table 1:** Baseline characteristics in study participants

	Olfactory-improvement N = 9, (%)	Olfactory-stable N = 15, (%)	Olfactory-decline N = 6, (%)	*P*-value
Female	7 (77.8)	9 (60.0)	2 (33.3)	0.227
Age, year	61.1 ± 6.8	55.1 ± 12.1	51.8 ± 9.2	0.188†
Duration of asthma, year	8.8 ± 5.4	9.9 ± 5.0	13.2 ± 6.4	0.259†
Smoking				0.358
Never-smoker	7 (77.8)	7 (46.7)	2 (33.3)	
Current-smoker	0 (0.0)	3 (20.0)	2 (33.3)	
Ex-smoker	2 (22.2)	5 (33.3)	2 (33.3)	
Allergic rhinitis	4 (44.4)	13 (86.7)	3 (50.0)	0.066
Chronic rhinosinusitis with or without nasal polyp	2 (22.2)	7 (46.7)	3 (50.0)	0.425
Hypertension	4 (44.4)	4 (26.7)	0 (0.0)	0.141
Diabetes mellitus	3 (33.3)	3 (20.0)	2 (33.3)	0.647
Biologics	4 (44.4)	3 (33.3)	2 (33.3)	0.449
Intranasal corticosteroids	2 (22.2)	8 (53.3)	5 (83.3)	0.67

^†^: *P*-value was obtained using one-way ANOVA

The Sino-nasal Outcome Test, Asthma Control Test scores, lung function parameters, sputum eosinophil levels, and fractional exhaled nitric oxide (FeNO) levels at both baseline and six months showed no significant difference among the three groups. Baseline blood eosinophil counts were also not significantly different among the three groups (348.30 ± 275.67 cells/uL vs. 426.83 ± 419.97 cells/uL vs. 679.11 ± 480.85 cells/uL). However, after six months, the blood eosinophil count was significantly higher in the olfactory-decline group (1021.61 ± 579.63 cells/uL) compared to the olfactory-improvement (282.99 ± 156.26 cells/uL, *P* = 0.008) and the olfactory stable (488.49 ± 456.70 cells/uL, *P* = 0.045) groups.

No significant differences were observed among the three groups regarding additional asthma maintenance therapies, including biologics and oral corticosteroids. During the study period, biologic therapies were administered to four patients in the olfactory-improvement group (dupilumab: n = 2, omalizumab: n = 1, reslizumab: n = 1), three patients in the olfactory-stable group (dupilumab: n = 2, omalizumab: n = 1), and two patients in the olfactory-decline group (dupilumab: n = 1, omalizumab: n = 1) (Table 2). Interestingly, blood eosinophil counts were decreased in the two patients treated with dupilumab in the olfactory-improvement group, whereas they increased in the patient treated with dupilumab in the olfactory-decline group. In the olfactory-stable group, one patient treated with dupilumab exhibited a decrease in blood eosinophil count, while another patient treated with dupilumab showed an increase in blood eosinophil count over the study period. Oral corticosteroids were maintained by two patients in the olfactory-improvement group and one patient in the olfactory-decline group throughout the study period.

**Table 2:** Treatment patterns of biologic therapy

Group	Biologics	Dose (mg)	Interval (weeks)	Total number of administrations	Treatment initiation
Olfactory- improvement	Omalizumab	300	4	8	Before study enrollment
	Dupilumab	300	4	6	At study enrollment
	Dupilumab	300	4 to 8	4	At study enrollment
	Reslizumab	100	4	6	At study enrollment
Olfactory- stable	Omalizumab	150	2	13	At study enrollment
	Dupilumab	300	4 to 6	6	At study enrollment
	Dupilumab	300	4 to 6	6	At study enrollment
Olfactory- decline	Omalizumab	300	2	13	At study enrollment
	Dupilumab	300	4	3	After study enrollment

We compared the changes in inflammatory biomarkers over six months among the olfactory-improvement, olfactory-stable, and olfactory-decline groups (Fig. 1B-D). The mean change in blood eosinophil counts differed across the three groups, with a decrease observed in the olfactory-improvement and olfactory-stable groups and an increase observed in the olfactory-decline group (− 30.0 ± 188.6 cells/µL vs. − 16.2 ± 258.4 cells/µL vs. 342.5 ± 305.3 cells/µL) (Fig. 1B). The olfactory-decline group exhibited statistically significant differences compared to both the olfactory-improvement group (*P* = 0.011) and the olfactory-stable group (*P* = 0.015). However, there were no significant differences in the changes in sputum eosinophil levels (− 8.6 ± 60.6 cells/µL vs. 29.4 ± 55.4 cells/µL vs. 29.6 ± 40.7 cells/µL) (Fig. 1C) or FeNO levels (− 29.8 ± 51.5 ppb vs. − 7.6 ± 26.7 ppb vs. − 3.75 ± 32.4 ppb) (Fig. 1D) among the groups (all *P* > 0.05).

The degree of eosinophilic infiltration in the olfactory cleft of patients with CRS is directly associated with the occurrence or recurrence of olfactory dysfunction, [8] and blood eosinophil counts are linked to olfactory impairment [9]. Although the underlying mechanism has not been fully elucidated, these findings suggest that eosinophilic inflammation may contribute to the pathophysiology of olfactory loss in CRS. In the present study, olfactory dysfunction was observed even in patients with moderate to severe asthma who did not have CRS or AR. Moreover, although no significant differences were observed in the prevalence of upper airway diseases among the three groups, changes in olfactory status were associated with alterations in blood eosinophil levels. These results suggest that systemic eosinophilic inflammation may be related to olfactory function in asthma.

The primary limitation of this study is the small sample size. Localized samples from the sinonasal mucosa or secretions were also not collected. Contrary to previous reports suggesting that biologics improve olfactory function, [10] our study found no significant effect of biologics in improving olfactory function. It may be attributed to instances where the administration of biologics was below the recommended dosage or the treatment duration was insufficient. Although biologic therapy may be a potential confounder, the lack of statistical adjustment for this factor may represent a limitation of the study. Therefore, the observed trends should be interpreted with caution. Additionally, the olfactory function was assessed using the YSK test, which is population-specific; therefore, the results may have limited generalizability outside Korean populations.

In conclusion, changes in blood eosinophil levels may be associated with alterations in olfactory function in asthma, and further study will be needed in the future.

## Data Availability

The datasets analyzed during the current study are available from the corresponding author on reasonable request.

## Abbreviations

AR: Allergic rhinitis
CRS: Chronic rhinosinusitis
FeNO: Fractional exhaled nitric oxide
NP: Nasal polyp
YOF: YSK Olfactory Function Test
